# Effects of sequential inhibitory and facilitatory repetitive transcranial magnetic stimulation on neurological and functional recovery of a patient with chronic stroke: A case report and literature review

**DOI:** 10.3389/fneur.2023.1064718

**Published:** 2023-01-27

**Authors:** Nan Chen, Xiao Qiu, Yan Hua, Jian Hu, Yulong Bai

**Affiliations:** Department of Rehabilitation Medicine, Huashan Hospital, Fudan University, Shanghai, China

**Keywords:** stroke, motor recovery, transcranial magnetic stimulation, neuroplasticity, case report

## Abstract

**Background and purpose:**

The effects of conventional protocols of repetitive transcranial magnetic stimulation (rTMS) in the chronic phase of stroke are limited. This study aimed to apply the sequential inhibitory and facilitatory rTMS for upper limb motor dysfunction post-stroke to observe the efficacy and explore the possible neurophysiological mechanism. We hypothesize that this protocol would both enhance the excitability of affected M1 and promote connections among motor areas.

**Case description:**

We reported a 55-year-old female patient with a 1-year chronic stroke and right-sided hemiplegia, who underwent the 14-session rTMS with seven sessions of low frequency (LF) and with seven sessions of high frequency (HF). Clinical scales mainly including Fugl-Meyer Assessment of Upper Extremity (FMA-UE), Action Research Arm Test (ARAT), neurophysiological measures, and functional near-infrared spectroscopy (fNIRS) were assessed before (T0), at the midpoint (T1), and after the intervention (T2).

**Outcomes:**

The patient exhibited post-intervention improvement in upper extremity function. There was increased excitability in the ipsilesional hemisphere and the opposite in the contralesional hemisphere. The interhemispheric inhibition (IHI) ratio increased from 2.70 to 10.81 and finally decreased to 1.34. Oxy-Hb signal was significantly decreased in affected M1 and mildly decreased in unaffected M1, while that of PMC and SMA on the affected side increased significantly.

**Conclusion:**

The sequential inhibitory and facilitatory rTMS significantly promoted motor recovery in the patient. Related mechanisms include upregulation of excitability in the ipsilesional hemisphere, return of interhemispheric balance, and neuroplasticity-induced cortical reorganization.

## Introduction

Stroke is an important cause of mortality and disability in adults (1), which places a heavy burden on families and societies around the world (2). Motor impairment is one of the most common complications post-stroke. More than half of the survivors with an initial paretic upper limb will still have problems with arm function months to years after their stroke (3), largely damaging activities of daily living. Repetitive transcranial magnetic stimulation (rTMS) is one of the non-invasive electrophysiological methods for the treatment of hemiplegia post-stroke, which can promote cortical reorganization and synaptic plasticity (4). Based on the interhemispheric competition model, numerous studies demonstrated that inhibition of the contralesional hemisphere by low-frequency rTMS (LF-rTMS; <1 Hz) (5) or facilitation of the ipsilesional hemisphere by high-frequency rTMS (HF-rTMS; >5 Hz) (6) can significantly improve upper limb motor function in patients with post-stroke (7–9), but the effects of conventional protocols in the chronic phase are limited (10). Patterned or complex coupled stimulation protocols might potentiate the efficacy of stimulation (11).

In this study, we introduce a coupled treatment initiated with seven-session 1 Hz rTMS over the contralesional primary motor cortex (M1) and followed by seven-session 10 Hz rTMS over the ipsilesional M1. Previous studies reported similar protocols in patients with subacute and chronic stroke with promising results (12, 13). On the one hand, this protocol could inhibit the excitability of the unaffected hemisphere at low frequency and thus enhance the excitability of M1 on the affected hemisphere based on the theory of transcallosal inhibition (14, 15). On the other hand, 10 Hz-rTMS has been shown to induce a long-lasting increase in glutamatergic synaptic strength, accompanied by structural remodeling of dendritic spines (16), thereby promoting the connection between affected M1 and the premotor cortex (PMC), the supplementary motor area (SMA), the primary motor cortex (M1), and other brain areas (17, 18) and regulating the neuroplasticity of the affected hemisphere. We hypothesize that the efficacy of stimulation could be potentially enhanced with this protocol. The sequential rTMS was applied to a 55-year-old woman with a chronic stroke of 1 year with a satisfying treatment effect; we now report the case below and explore the possible neurophysiological mechanism through functional near-infrared spectroscopy (fNIRS) and transcranial magnetic stimulation (TMS) assessments.

## Case description

The Ethics Committee of Huashan Hospital affiliated with Fudan University (No. KY2021-1005) approved the study protocol and intervention, and informed consent was obtained from the patient before enrolling in the study.

The 55-year-old female patient was right-handed and graduated from senior high school. She was retired with good financial and family support. She has a history of hypertension, type 2 diabetes, and coronary heart disease, controlled by daily medication. There was no relevant genetic or psychosocial history in her family. At the time of stroke onset in January 2021, she presented with complete right hemiplegia without unconsciousness, and then, she was immediately transferred to the local District Central Hospital in Shanghai. Her blood pressure was 140/80 mmHg and her heart rate was 80 beats per min. Based on diffusion-weighted imaging (DWI) of magnetic resonance imaging (MRI) with a high-intensity area in the left basal ganglia, the patient was diagnosed with cerebral infarction and received conservative antiplatelet therapy and butylphthalide infusion therapy to improve cerebral circulation. One-hour bedside rehabilitation was offered to her every day. When she was discharged 12 days after onset, she had a Brunnstrom Staging (BS) of 1/1/3 (upper extremity/hand/lower extremity) in her right extremity, with a muscle strength of 0 in the upper extremity and 1–3 in the lower extremity. She was able to stand and walk with support but was unable to initiate movement in the paretic upper limb. Then, the patient was transferred to a general rehabilitation hospital for multidisciplinary rehabilitation including limb positioning, passive stretching, sit-to-stand, muscle strength exercises, balance training, hand function training, acupuncture, massage, electrical stimulation, and so on. The recovery in the affected upper limb continued gradually along a proximal-distal mode. During the enrolment in March 2022, she could flex and extend her elbow obviously and flex weakly but not extend her fingers on the affected side. Muscle strength of the right upper limb was grade 3/3/2/2 (shoulder flexion/elbow flexion/wrist flexion/finger flexion), and muscle tone was grade 1/1+/1 (shoulder adduction/elbow flexion/wrist flexion), with BS reaching 3/3/3.

## Intervention

rTMS were conducted using MagTD (YIRUIDE Company, Wuhan, China) connected with a 90-mm figure-of-eight coil. The coil was positioned tangentially on the scalp with the handle pointing 45° posterolaterally. Stimulation intensity was gradually increased, and coil position was shifted slightly until we determined the optimal stimulation site (“hot spot”) where the largest motor-evoked potential (MEP) could be consistently elicited from the contralateral abductor pollicis brevis (APB) (9, 19). The “hot spot” served as the target for the rTMS modulation. The stimulation intensity was set at 120% of the resting motor threshold (RMT) of APB (7, 20). If stimulus intensity exceeds maximal stimulator output (MSO), 100% MSO will be adopted. The patient accepted seven-session 1 Hz rTMS (a 20-min train of 1 Hz rTMS, a total of 1,200 pulses in one session) over the non-lesional hemisphere (13, 21) followed by seven-session 10 Hz rTMS (9, 22, 23) (1-s trains of 10 Hz with 9-s inter-train intervals over 12 min, total 1,200 rTMS pulses in one session) over the lesional hemisphere. Besides, she also received conventional rehabilitation programs, such as task-oriented training, range-of-motion exercise, muscle exercise, gait training, and acupuncture, for 150–180 min daily during the study period. Medical treatment was also provided to her, including dapagliflozin, insulin lispro, and insulin glargine for blood sugar control, losartan and levamlodipine for blood pressure control, aspirin and clopidogrel for antiplatelet therapy, and atorvastatin for lowering lipid levels.

## Assessments

### Clinical outcome measures

Assessments were acquired at baseline (day 0, T0), in the middle of the intervention (day 7, T1), and after treatment (day 14, T2). Clinical scales included the Fugl-Meyer Assessment of Upper Extremity (FMA-UE), the Action Research Arm Test (ARAT), the Modified Ashworth Scale (MAS), the BS, the Barthel Index (BI), and the Mini-Mental Score Examination (MMSE).

### Neurophysiological measures

Motor cortical excitability was evaluated by single-pulse TMS, and interhemispheric inhibition (IHI) was evaluated by paired-pulse TMS. First, we tested the RMT, which was defined as the lowest stimulus intensity that produced MEPs >50 μV in at least five out of 10 trials when the target muscle (APB) was at rest (24). MEP amplitude and latency were measured as peak–peak (μV) of the mean MEP and the time (ms) from the onset of the stimulus to the beginning of the MEP, respectively. Central motor conduction times (CMCT) were calculated by deducting the peripheral conduction time (PMCT) from MEP latency, and PMCT was obtained by stimulating the brachial plexus. When it comes to IHI, we delivered a conditioning pulse (110% RMT) to the hotspot of one hemisphere followed 10 ms later by a test pulse (120% RMT) to the hotspot on the opposite hemisphere. Besides, a single test pulse to the target hemisphere was also delivered, and we calculated the ratio of the average amplitude of paired-pulse MEPs to the average amplitude of single-pulse MEPs for both hemispheres, expressed as IHI_Ipsi−to−Contralesional_ and IHI_Contra−to−Ipsilesional_, respectively (25, 26). The lower the value, the stronger the IHI. IHI ratio was determined by the inhibition of the ipsilesional to the contralesional hemisphere divided by that of the contralesional to the ipsilesional hemisphere, which was defined as: IHI ratio = (1 − IHI_Ipsi−to−Contralesional_)/(1 – IHI_Contra−to−Ipsilesional_) (26). This ratio provided us with a normalized and quantitative parameter to assess the nature of IHI, and the ratio of >1 implied larger inhibition from the affected to unaffected hemisphere compared to that from the unaffected to affected hemisphere. The above operations were repeated 10 times each.

### fNIRS data acquisition and analysis

fNIRS data were acquired using a 41-multichannel fNIRS instrument (BS-3000, Wuhan Union Technology Co., Wuhan, China). The fNIRS data were sampled with a frequency of 20 Hz. A customized brain cap consisting of 32 probes (16 sources and 16 detectors) was placed on the head of the patient. Referring to the international EEG 10–20 system, all the source probes and detector probes were, respectively located over the bilateral prefrontal cortex (PFC), the M1, the premotor cortex (PMC), the supplementary motor area (SMA), and the Broca's area, constituting 41 channels. Data were recorded at wavelengths of 690 and 830 nm. Here, we used a block paradigm design consisting of 60-s rest at baseline, 20-s task, and 20-s rest three times. The patient was instructed to sit in a comfortable position in a chair with the upper extremities relaxed at rest condition. During the task, the patient was asked to perform repetitive movements of flexion and extension of the paretic elbow at a comfortable speed, with 1 kg carried on the forearm (27).

The fNIRS data were exported to MATLAB (R2013a, MathWorks, USA) for further data processing and analysis, and the HbO_2_ signal was chosen as the marker of neural activity in the study. The data were analyzed in Homer2 (28). The raw fNIRS signals were first transferred into hemodynamic signals according to the modified Beer–Lambert law. After removing the invalid channels, the visible motion artifacts, and physiological noise, a Butterworth band-pass filter between 0.01 and 0.1 Hz was applied to filter the HbO_2_ signals to eliminate slow drift and cardiac pulsation. To identify the task-related cortical activation, the changes in concentration of HbO_2_ (ΔHbO_2_) were computed as follows: ΔHbO_2_ = HbO_2task_ – HbO_2baseline_. HbO_2baseline_ was defined as the average value of the HbO_2_ signals at the last 10 s during the resting-baseline period. The HbO_2task_ was defined as the average value of the HbO_2_ signals derived from a 6-s window around the peak of the most positive deflection within the 20 s following task onset (29). The ΔHbO_2_ of all channels in each cortical area was averaged to represent cortical activation. To evaluate the interhemispheric asymmetry of cortical activation, we calculated the laterality index (LI) of each cortical region (30). The values of LI were between −1 and 1, with positive LI indicating greater activation in the ipsilesional than in the contralesional hemisphere and vice versa. LI was calculated as follows:


$$\begin{array}{l} \text{LI}=(\text{Δ}{\text{HbO}}_{\text{2}}\;\left(\text{ipsilesional hemisphere}\right)\\ \;\;-\text{Δ}{\text{HbO}}_{\text{2}}\;\left(\text{contralesional hemisphere}\right))\\ \text{ /}(\text{Δ}{\text{HbO}}_{\text{2}}\;\left(\text{ipsilesional hemisphere}\right)\\ \;\text{ + Δ}{\text{HbO}}_{\text{2}}\;\left(\text{contralesional hemisphere}\right)). \end{array}$$

## Outcomes

The test was smooth and the patient completed the 14-day rTMS sessions without adverse side effects during the treatments and assessments.

### Clinical scores

Clinical scales for the patient are presented in Table 1. The scores of both the FMA-UE and ARAT showed progress at both measurement points after starting treatment. Spasticity relief was demonstrated from MAS value from 1 to 0 at shoulder-joint and 1+ to 1 at elbow-joint with no change for hand. BI increased by 5 points from T1 to T2, and the patient could walk independently without any assistance. Notably, the BS of the hand progressed from level 3 to 4, and her thumb could be slightly pinched and loosened with the remaining four fingers extended actively in a small range. In addition, the patient's MMSE score remained at 30 and she had good cognition throughout the treatment.

**Table 1:** Details of clinical scales.

**Item**	**T0**	**T1**	**T2**	**Change (T1–T0)**	**Change (T2–T0)**
FM-UE	17	19	23	2	6
ARAT	3	6	9	3	6
MAS-shoulder	1	0	0	−1	−1
MAS-elbow	1+	1+	1	0	−0.5
MAS-hand	1	1	1	0	0
BS-UE	3	3	3	0	0
BS-HAND	3	3	4	0	1
BI	85	85	90	0	5
MMSE	30	30	30	0	0

T0, baseline; T1, day 7; T2, day 14; FM-UE, Fugl-Meyer of Upper Extremity; ARAT, Action Research Arm Test; MAS, Modified Ashworth-Scale; BS, Brunnstrom stages; UE, lower extremity; BI, Barthel Index; MMSE, mini-mental state examination.

### Neurophysiological measures

We elicited MEPs of the affected limb from the ipsilesional hemisphere of this patient. As illustrated in Table 2, ipsilesional RMT and MEP latency decreased, and MEP amplitude increased after treatment. There was little change in ipsilesional CMCT. As for the contralesional hemisphere, the RMT was increased by 10% at the mid-intervention and returned to 50% post-therapy. The values of MEP latency and CMCT decreased slightly at T1 and increased significantly after treatment beyond the initial value. MEP amplitude was decreased by 126.45 μV from T0 to T1 and increased by 58.45 μV to T2. Excessive IHI from the ipsilesional to the contralesional hemisphere was observed at baseline, and the value did not change significantly throughout treatment. IHI_Contra−to−Ipsilesional_ increased at T1 and decreased significantly at T2, even below baseline, which led to an increase in the IHI ratio from 2.70 to 10.81 and finally to 1.34.

**Table 2:** Changes in neurophysiological measures and cortical asymmetry as evaluated by fNIRS.

**Item**	**T0**	**T1**	**T2**	**Change (T1–T0)**	**Change (T2–T0)**
**Neurophysiological measures**					
**AH**					
MEP amplitude (μV)	112.44	105.27	124.36	−7.17	11.92
MEP latency (ms)	29.24	27.53	28.07	−1.71	−1.17
RMT (%)	95%	90%	90%	−5	−5
CMCT (ms)	13.39	13.17	13.45	−0.22	0.06
**UH**					
MEP amplitude (μV)	245	118.55	177	−126.45	−68
MEP latency (ms)	23.62	22.05	25.95	−1.57	2.33
RMT (%)	50%	60%	50%	10%	0
CMCT (ms)	8.14	6.83	11.63	−1.31	3.49
**IHI**					
IHI_Ipsi−to−Contralesional_	0.19	0.13	0.24	−0.06	0.05
IHI_Contra−to−Ipsilesional_	0.7	0.92	0.43	0.22	−0.27
IHI ratio	2.7	10.81	1.34	8.11	−1.36
**LI**					
M1	−0.05	/	−0.49	/	−0.44
PMC	0.28	/	0.58	/	0.3
SMA	−0.14	/	0.88	/	1.02

T0, baseline; T1, day 7; T2, day 14; fNIRS, functional near-infrared spectroscopy; AH, affected hemisphere; UH, unaffected hemisphere; MEP, motor evoked potential; RMT, resting motor threshold; CMCT, corticomotor conduction time; IHI, interhemispheric inhibition; Ipsi, ipsilesional; Contra, contralesional; LI, laterality index; M1, primary motor cortex; PMC, premotor cortex; SMA, supplementary motor area.

### Activation of cortical core motor regions

**Figure 1:**
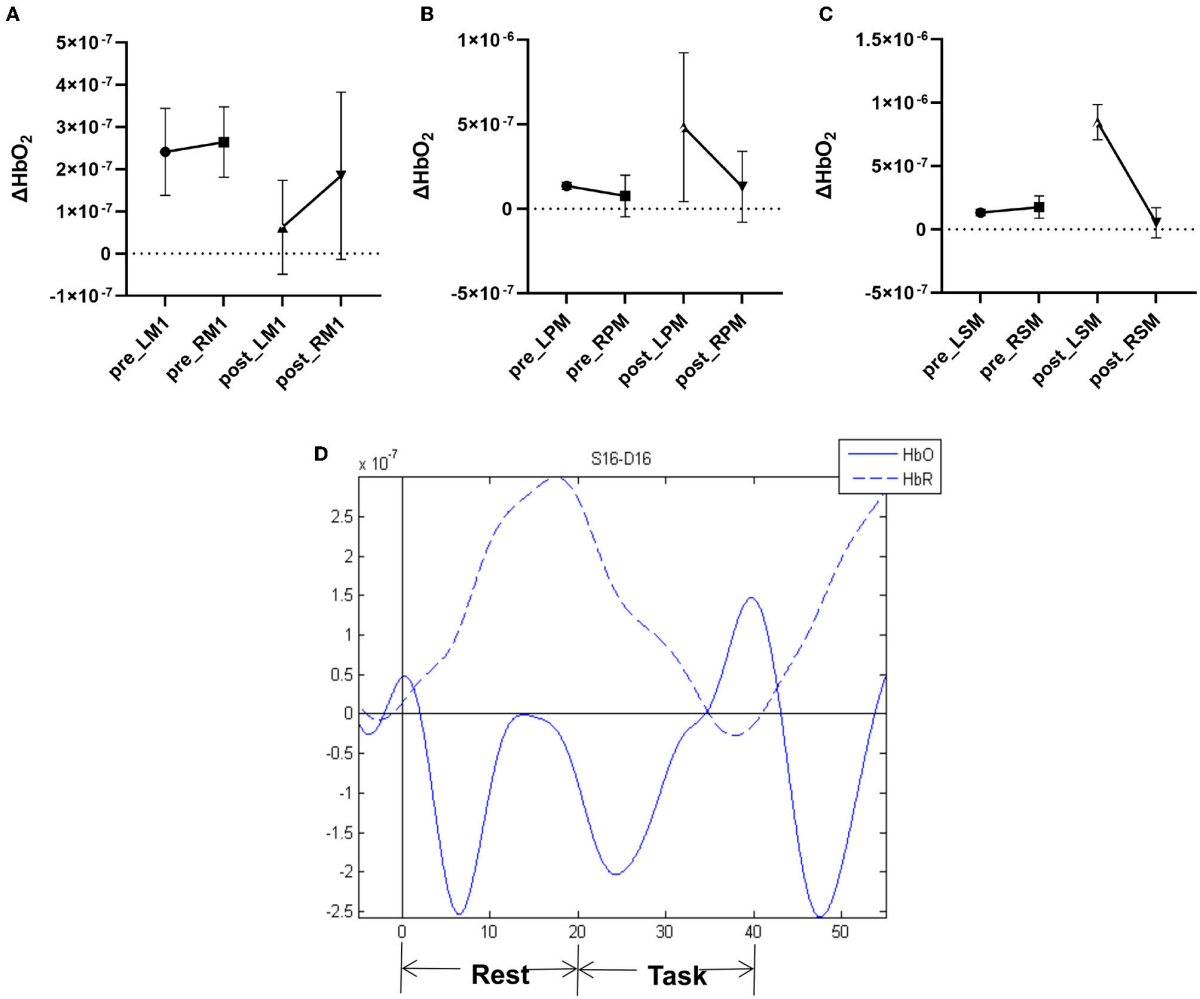
Pre- and post-therapy oxygenated hemoglobin (oxy-Hb) concentration measured by fNIRS during movement of the paretic upper limb in **(A)** M1 primary motor cortex, **(B)** PM premotor cortex, and **(C)** SMA supplementary motor area. **(D)** Example of hemodynamic response function (HRF) time series.

At baseline, the patient exhibited activation of bilateral motor cortices during the movement of the paralyzed upper extremity (Figure 1, Table 3). After 14-day treatment, the oxy-Hb signal was significantly decreased in affected M1 and mildly decreased in unaffected M1, which resulted in a decrease in LI of M1. However, cortical activation showed a significant increase in affected PMC and SMA, increasing the LI of PMC and SMA.

**Table 3:** Changes of HbO_2_ concentration in cortical core motor regions.

**Channels**	**Cortical region**	ΔHbO_2_ (*10^−6^) (T0)	ΔHbO_2_ (*10^−6^) (T2)
28, 29, 32	Ipsilesional M1	0.241	0.063
27, 30	Ipsilesional PMC	0.136	0.484
31, 33	Ipsilesional SMA	0.132	0.846
34, 35, 36	Contralesional M1	0.264	0.185
40, 41	Contralesional PMC	0.077	0.130
38, 39	Contralesional SMA	0.176	0.052

T0, baseline; T2, day 14; M1, primary motor cortex; PMC, premotor cortex; SMA, supplementary motor area.

## Discussion

Two weeks of sequential inhibitory and facilitatory rTMS significantly facilitated motor performance and recovery in the patient with chronic subcortical stroke. Related mechanisms include upregulation of excitability in the ipsilesional hemispheric, return of interhemispheric balance, and neuroplasticity-induced cortical reorganization.

### The cortical reorganization in ipsilesional PMC and SMA and contralesional M1

In our study, the MEPs of the APB could be elicited on the affected side indicating that the structure of ipsilesional CST was reserved. After the 14-day intervention, there was increased excitability in the affected hemisphere. In the fNIRS assessment, we found decreased activation of ipsilesional M1 and significantly increased activation of ipsilesional PMC and SMA during the movement of the paretic upper extremity after treatment. The stimulus intensity we took on ipsilesional M1 was close to MSO, and the site we stimulated in the affected hemisphere was probably the premotor areas, thereby activating the CST from those areas and causing some of the effects we observed.

Functionally, PMC and SMA are involved in motor planning, control, and learning, as they project to M1 for movement execution (31–33). In patients with significant functional disruption of the corticospinal system, task-related brain activation shifts from primary to secondary motor networks (34). Our findings align well with previous studies showing that the plastic reorganization of ipsilesional PMC and SMA contributes to functional recovery (35–38). The abovementioned phenomenon can be explained by the mechanism that movements might be directly controlled by the increased corticospinal pathways originating from PMC and SMA (39–42). Fridman et al. (43) reported that, in well-recovered chronic stroke patients with lesions located in the internal capsule, motor potentials evoked by TMS stimulation of the ipsilesional PMC were larger and of shorter latency than those evoked by stimulation of the ipsilesional M1. Subsequently, an fMRI study (39) confirmed that the integrity of the PMC-derived CST correlates with grip strength.

In the contralesional hemisphere, RMT increased at T1 and decreased to baseline at T2, indicating a gradual return to normal excitability after HF-TMS, but increased contralesional CMCT and MEP latency and decreased MEP amplitude suggested decreased excitability compared to baseline. Considering that there was a clear decrease in LI of M1 in the fNIRS assessment, we speculate that for this patient, the ipsilateral CST derived from the contralesional M1 was considered as a possible mechanism for motor improvement (44, 45). Even in a subset of well-recovered patients, activation of contralesional M1 was effective for motor recovery (46). Patients with more severe impairments of ipsilesional CST might rely more on contralesional hemisphere activity (47).

Taken together, effective functional recovery should fully utilize ipsilesional and contralesional resources, and the cortical reorganization in ipsilesional PMC and SMA and contralesional M1 played an important role in the recovery of motor function throughout the intervention. As for the different changes in the IL of M1 and PMC/SMA, we considered that it might be due to the limited CST originating from ipsilesional M1. Sequential rTMS would lead to neural remodeling in ipsilesional PMC/SMA to promote functional recovery, and the increased activation of ipsilesional PMC/SMA might contribute to abnormally increased neural activity in the contralesional motor areas (48).

### Restoration of interhemispheric balance

It is noteworthy that, at baseline, IHI_Ipsi−to−Contralesional_ was much lower than IHI_Contra−to−Ipsilesional_, suggesting that the affected M1 had a stronger inhibitory effect on the healthy M1, and we consider it as a maladaptive process. After the 14-day sequential rTMS over affected M1, there was a significant decrease in IHI_Contra−to−Ipsilesional_ and a slight increase in IHI_Ipsi−to−Contralesional_, reducing the IHI ratio from 2.70 to 1.34 and leading to a balance between two hemispheres. It has been demonstrated that chronic patients with more impairment (FM-UE ≤ 43) have stronger IHI from the contralesional to the ipsilesional hemisphere with greater motor performance, while patients with less impairment (FM-UE > 43) show the opposite (49). The former is manifested in our patients. The negative impact of the ipsilesional motor areas on the contralesional M1 has been confirmed to normalize gradually with functional recovery (50). In combination with TMS and electroencephalography (EEG), Casula et al. (51) have found that in patients with chronic stroke, the better the strength of the affected hand is restored, the closer the interhemispheric balance is to 1. Combined with the abovementioned studies and this case, we can conclude that the better the recovery of motor function, the more stable balance between the two hemispheres.

### The advantages of sequential rTMS

A previous randomized controlled study has shown that 10 sessions of 1 Hz rTMS followed by 10 sessions of iTBS could improve FMA-UE of patients with chronic stroke by about three points (12), which was lower than the increase in this case. rTMS can maximize metaplasticity effects to induce or restore synaptic plasticity (52). HF-rTMS and LF-rTMS can result in strengthening (long-term potentiation/LTP) or weakening (long-term depression/LTD) of synaptic connections and efficacy, respectively (53), and the efficacy of LTD or LTP depends on the integrity of the corticospinal pathway (54). Therefore, priming the intact hemisphere first would be more conducive to promoting synaptic plasticity. LF-rTMS over unaffected M1 can effectively reduce RMT and increase MEP amplitude in unstimulated M1 (15). The increased cortical excitability in the lesional hemisphere might be related to the increased intrinsic excitability of the excitatory interneurons responsible for glutamatergic non-NMDA receptors, and these changes are likely mediated by interhemispheric callosal connections (55). In addition, the effects of LF-rTMS can be continued into the next session, thus enhancing the effectiveness of subsequent high-frequency transcranial magnetism (12), as reflected in the FM-UE and ARAT results and distal upper limb function. Compared to LF-rTMS, HF-rTMS over the ipsilesional M1 could be more conducive to the functional connectivity reorganization of the ipsilesional motor network (17). Moreover, HF-rTMS can enhance the interhemispheric connection both anatomically and functionally (56–58), providing the basis for restoring interhemispheric inhibitory balance regardless of which hemisphere has a stronger IHI.

## Conclusion

In this study, we have proposed a protocol of sequential inhibitory and facilitatory rTMS that significantly improved motor performance in the patient with chronic subcortical stroke, and explored the neurophysiological mechanism through fNIRS and TMS. Further randomized controlled trials are needed to confirm the effectiveness of sequential rTMS for motor function improvement in patients with chronic phase and to explore possible underlying mechanisms of interhemispheric balance and cortical reorganization.

## Limitations

First, the study lacked fNIRS mid-term assessment and long-term follow-up of the patient, so we are unsure about the cortical activation after LF-TMS and the duration of the effect after treatment. Second, we did not use DTI to assess the structural integrity of the pyramidal tract and the contribution of the CSTs from ipsilesional premotor areas and contralesional M1 to motor recovery.

## Data availability statement

The original contributions presented in the study are included in the article/Supplementary material, further inquiries can be directed to the corresponding authors.

## Ethics statement

The studies involving human participants were reviewed and approved by the Ethics Committee of Huashan Hospital affiliated to Fudan University. The patients/participants provided their written informed consent to participate in this study. Written informed consent was obtained from the individual(s) for the publication of any potentially identifiable images or data included in this article.

## Author contributions

NC did the intervention, collected data, and wrote the paper. XQ analyzed the data. YH evaluated the patient. JH and YB revised the literature and offered the grant support. All authors contributed to the article and approved the submitted version.
