# Supplementary material for: Effects of sequential inhibitory and facilitatory repetitive transcranial magnetic stimulation on neurological and functional recovery of a patient with chronic stroke: A case report and literature review
Source: Front Neurol. 2023 Jan 27;14:1064718. doi: 10.3389/fneur.2023.1064718 (PMC9911674; doi:10.3389/fneur.2023.1064718)
Supplement: Supplementary file 1 [file Data_Sheet_1.PDF]

January 2021
(Stroke onset)

Complain: Sudden right-sided limb weakness
BP 140/80 mmHg, Pulse rate 80/min, right limb power 0/5, right Babinski sign (+)
MRI: a high-intensity area in the left basal ganglia
Diagnosis: cerebral infarction & hypertension & type 2 diabetes & coronary heart disease
Treatment: Medication and physiotherapy

12 days later
(To a general rehabilitation hospital)

Right upper limb power 0, lower limb power 1-3, BS 1/1/3
Rehabilitation treatment: limb positioning, passive stretching, sit to stand, muscle strength exercises, balance training, hand function training, acupuncture, massage, electrical stimulation etc.

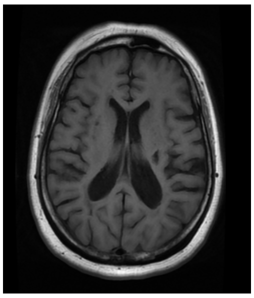

March 2022
(Enrolment)

Right upper limb power 3/3/2/2 (shoulder flexion/ elbow flexion/ wrist flexion/ finger flexion)
MAS 1/1+/1 (shoulder adduction/ elbow flexion/ wrist flexion)
BS 3/3/3, FM-UE 17, ARAT 3, BI 85, MMSE 30

Figure1. Timeline with relevant data of the case

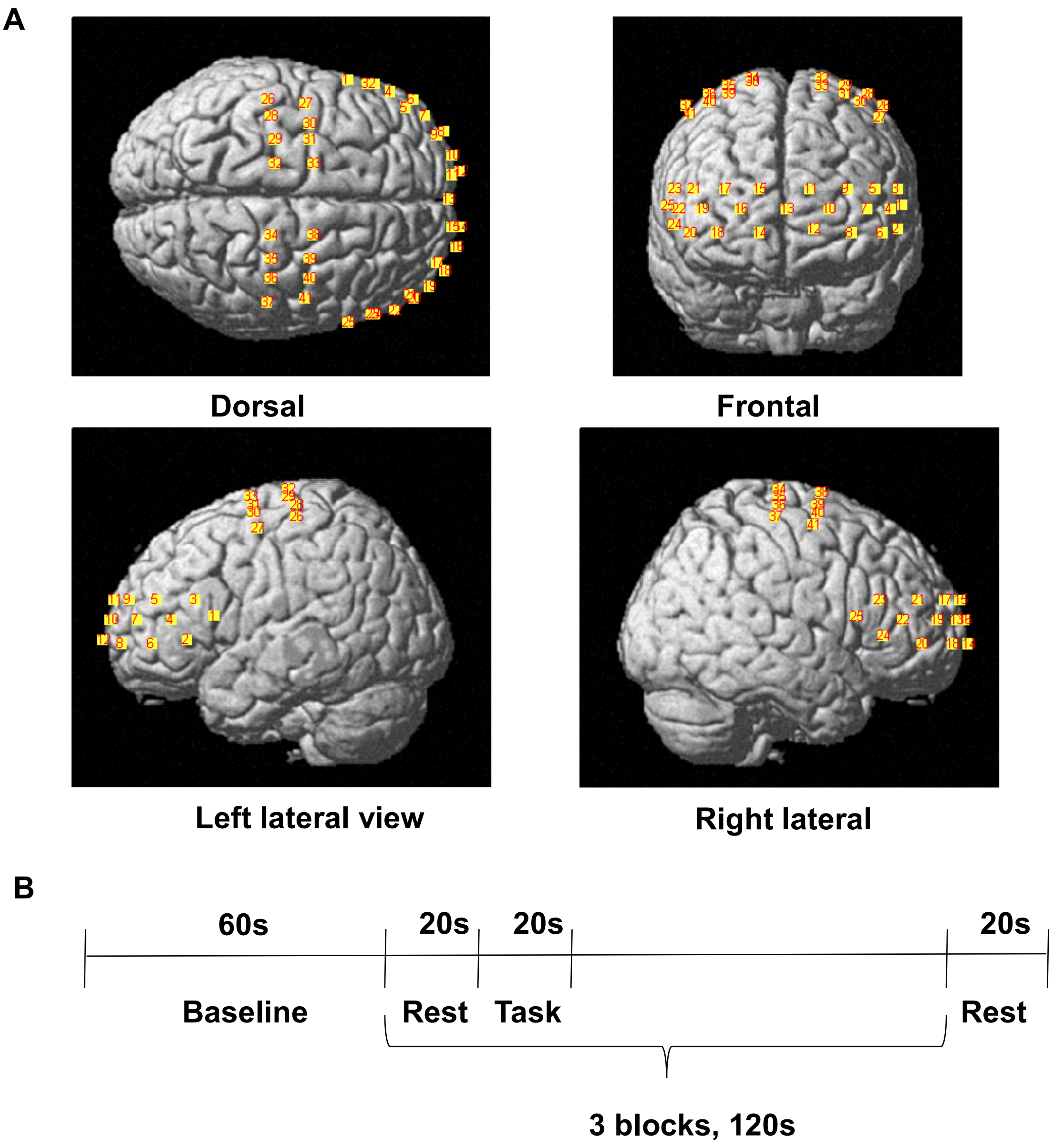

Figure 1. (A) fNIRS 41-channel placement. (B) The fNIRS experimental setup.
